# Recycle Option for Municipal Solid Waste Incineration Fly Ash (MSWIFA) as a Partial Replacement for Cement in Mortars Containing Calcium Sulfoaluminate Cement (CSA) and Portland Cement to Save the Environment and Natural Resources

**DOI:** 10.3390/ma17010039

**Published:** 2023-12-21

**Authors:** Nikolina Poranek, Jan Pizoń, Beata Łaźniewska-Piekarczyk, Adrian Czajkowski, Ruslan Lagashkin

**Affiliations:** 1 Department of Technologies and Installations for Waste Management, Faculty of Energy and Environmental Engineering, The Silesian University of Technology, Konarskiego 18, 44-100 Gliwice, Poland; 2 Department of Building Engineering and Building Physics, Faculty of Civil Engineering, The Silesian University of Technology, Akademicka 5, 44-100 Gliwice, Poland; 3 Doctoral School, The Silesian University of Technology, Akademicka 2A, 44-100 Gliwice, Poland; 4 Department of Power Engineering and Turbomachinery, Faculty of Energy and Environmental Engineering, The Silesian University of Technology, Konarskiego 18, 44-100 Gliwice, Poland; 5 EnergySol s.c., Przepiórek 53, 43-100 Tychy, Poland; 6 Department of Electronics and Nanoengineering, Faculty of Nanoscience and Advanced Materials, Aalto University, Tietotie 3, 02150 Espoo, Finland

**Keywords:** municipal solid waste incineration fly ash (MSWIFA), circular economy, European Green Deal, Sustainable Development Goals, APCr—Air Pollution Control residue, calcium sulphoaluminate cement (CSA), Portland cement CEM I, eco-concrete

## Abstract

Reduction of emissions, energy consumption, and use of substitutes for natural resources is an element of sustainable development and the circular economy. Cement production is a process with a high carbon footprint; therefore, minimizing the use of this material has a significant impact on reducing environmental costs. A substitute for cement is municipal solid waste incineration fly ash (MSWIFA). The article presents a method of making an eco-concrete with the use of municipal solid waste incineration hazardous fly ash. The use of secondary waste for the production of building materials additionally contributes to achieving climate neutrality established by the European Union and China. The article analyzes the physicochemical properties of various MSWIFAs, the amount and leachability of heavy metals, and selected elements from MSWIFA and concrete properties. The technical properties of mortars containing MSWIFA were investigated. Consistency is not affected by MSWIFA content, although the workability time is prolonged. Air entraining admixture efficiency is lowered, but the effect lasts longer. The initial setting time is prolonged, and the flexural and compressive strengths are decreased in early terms because of the zinc presence in MSWIFA. MSWIFA does not influence the water demand, volume stability of mortars, or microstructure of cement’s hydration products.

## 1. Introduction

**Figure 1:**
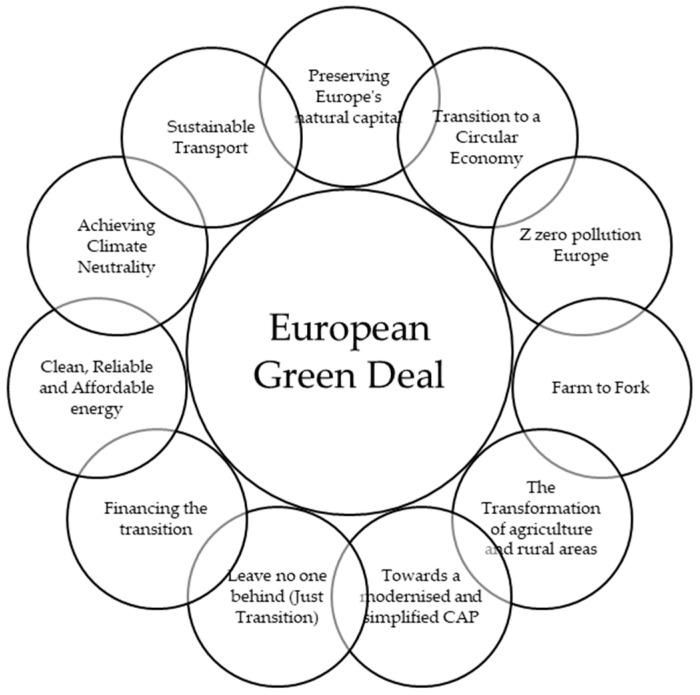
Priorities for the European Green Deal [3].

Europe’s goal is to become climate neutral by 2050, while China, the world’s second-largest economy, has plans to achieve the same goal by 2060 [1,2]. The first initiatives of the European Green Deal include the European Climate Law, the European Climate Pact, the 2030 Climate Goals Plan, and the EU Climate Change Adaptation Strategy, as shown in Figure 1.

The World Organization of the United Nations (UN) is in line with the objectives of the European Green Deal, which result from the adoption of the 2030 Agenda for Sustainable Development. The agenda was adopted by the General Assembly Resolution in 2015 by all 193 member states. It contains 17 Sustainable Development Goals (SDGs) and 169 targets [3,4]. Municipal solid waste incineration fly ash (MSWIFA) management is connected mainly with 12 and 13 SDGs. The SDGs are to be achieved by 2030 through implementation in five areas, called 5xP (people, planet, prosperity, peace, and partnership). MSWIFA management is connected with the planet.

Both the UN and the European Union are consistent with the definition of the threat posed by 2 °C global warming from before the pre-industrial era. The European Union promotes the encouragement to limit the temperature rise in the 2030 Climate Targets Plan, while the parties to the UN Framework Convention adopted a position in 2009 in Copenhagen to act to prevent temperatures from rising above a critical value. Increasing the global average temperature (according to both sides) is directly related to anthropogenic activity. The solution is to achieve zero net greenhouse gas emissions to the atmosphere, which is related to, among others, the change from a linear to a circular economy [5,6,7].

From the circular economy point of view, it is relevant to reduce the energy consumption of the industry, minimize waste and recycle it, develop technology and cleaner production, extend products and material life (Life Cycle Assessment), and look for environmentally safer solutions for planning, production, and waste management [4,5,8,9,10].

In 2015, the European Commission introduced “Closing the loop—An EU action plan for the Circular Economy”, which presents a specific program and priorities for environmental protection. The most relevant areas of introducing a circular economy include production, consumption, waste management, the expansion of the market that uses secondary (recycling) resources, the introduction of innovations and new investments, and the entire process of monitoring. The priority areas are plastics, food waste, critical raw materials, construction and demolition, and biomass [11]. In the linear economy, landfilling was the most common form of waste management. Due to the lack of regulation on the market, this form was also the cheapest solution. However, landfilling is not included in the circular economy, and in Directive 2008/98/EC of the European Parliament, in Article 4. Landfilling is in last place in the waste management hierarchy [6,12,13].

### 1.1. Municipal Solid Waste Incineration Fly Ash Challenges

Thermal processing is an integral part of waste management and the most hygienic method of waste disposal. Installations for thermal treatment of municipal waste (Waste-to-Energy installation, WtE) are currently the only solution on the market for the management of contaminated or repeatedly processed waste that has lost its physicochemical properties and quality [14,15]. These include, for example, dirty, greasy packaging and leftovers (residue or mixed waste) and repeatedly processed materials such as paper or plastic. In WtE, it is possible to recover heat and electricity from batch waste. The current exhaust gas treatment systems are of high quality, and BAT (Best Available Techniques/Technology) systems are introduced, which meet the high requirements of the Directive 2010/75/EU of the European Parliament and the Council on industrial emissions (the Industrial Emissions Directive, or IED) [16]. Sulfur compounds, heavy metals, polychlorinated dibenzo-p-dioxins, and furans are captured in the exhaust gas treatment system on filters, usually bag filters. Unfortunately, after cleaning the exhaust gases, hazardous fly ash (APCr—Air Pollution Control residue) is produced, which is currently stored. APCr, as a MSWIFA, can be solidified in the stabilization and solidification process (s/s) into monoblocks and placed in dedicated landfills. Usually, the landfills are located underground, and the monolith complements the mining void. Despite the function of strengthening the area, post-mining voids have a limited cubature, and storage is not included in the circular economy [17,18,19].

If the fly ash was entirely reused in industry, WtE would constitute more environmentally friendly installations. It is an example of the use of the high-tech industry in waste management, where the installations are based on the latest scientific achievements in the production process and the product itself. High-quality filters that minimize the plant’s emissions and its ongoing monitoring, as well as the complete closing of the cycle through the use of secondary waste, including hazardous waste, is a trend that is globally promoted and disseminated [12,20].

### 1.2. Place of Arising and Composition of Fly Ash in the Waste Incineration Plant and Flue Gas Cleaning System

Most combustion technologies require exhaust gas treatment, in particular municipal solid waste incineration plants. Modern, multi-stage flue gas treatment systems from thermal treatment of municipal waste can remove 95–99% of pollutants from the flue gas stream. The exhaust gas treatment system includes the following:

- Acid pollution reduction systems;
- Heavy metal, dioxin, and furan reduction systems;
- Nitrogen oxide removal systems;
- Exhaust gas dedusting system, which may consist of electrostatic precipitators, fabric filters, and cyclones.

**Figure 2:**
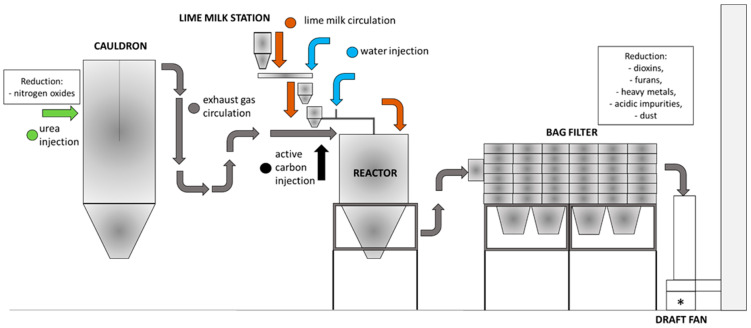
Scheme of flue gas treatment and formation of hazardous fly ash.

The tested fly ash comes from the exhaust gas treatment system, which is dedusted by using a fabric filter. The treatment of acidic pollutants is carried out using a semi-dry method (to reduce acidic pollutants). The semi-dry method is combined with the dust-blasting method, which uses activated carbon. Activated carbon is intended to reduce heavy metals as well as dioxins and furans. The amount of activated carbon used is approx. 2 kg per 24 Mg of the produced tested fly ash (European Waste Codes 19 01 07 *—solid wastes from gas treatment, *—hazardous waste). The primary method is used for the denitrification of flue gases. The secondary method aims to reduce NOx emissions by using the SNCR (Selective Non-Catalytic Reduction) method with the use of urea. Figure 2 shows a diagram of the flue gas treatment, which results in the formation of municipal solid waste incineration fly ash (MSWIFA).

MSWIFA is a hazardous waste with a high concentration of heavy metals. Table 1 and Table 2 present an overview of selected fly ash [21,22].

Table 1 and Table 2 show the results of the physicochemical tests. The tested fly ashes differ from each other. Fly ash properties depend on the season, municipal waste collection point, waste incineration process, flue gas cleaning system, and flue gas cleaning technology. Some of the properties depend on the chemical composition of the municipal waste that is incinerated. These include, e.g., chlorine, sulfur, and heavy metals. The carbon content depends on the time and temperature of combustion and the amount of active carbon dosed in the exhaust gas treatment. The amount of calcium depends on the amount of lime milk dosed in waste gas treatment [31]. The research shows that each batch of waste before use in the construction mixture should be tested to determine the ideal mix proportions and obtain the best immobilizing properties.

### 1.3. Concrete with MSWIFA as an Eco-Product

An alternative to the disposal of secondary waste is the production of a construction product. Concrete with MSWIFA is more environmentally friendly due to the lack of use or the use of a small amount of cement. The cement production process is highly emissive, energy-intensive, and uses natural resources. Carbon dioxide emission factors for Portland cement are the decarbonization process (48%), the firing process (42%), electricity (5%), and transport (5%). Producing 1 Mg of cement emits almost 1 Mg of carbon dioxide and consumes over 1 Mg of natural resources.

Pre-treatment can be performed to prepare the secondary waste for use in the final product. Pre-treatment aims to improve the physicochemical properties and/or reduce the leaching of compounds, i.e., heavy metals. Among other methods and waste preparation, it is possible to distinguish, i.e., ceramization, vitrification, chemical activation (NaOH, CaOH_2_, NA_2_SiO_3_ + NaOH, Na_2_CO_3_ + NaOH, NH_4_OH), acid treatment with dilute solutions (HCl, H_2_SO_4_), chemical stabilization (FeSO_4_, PO_4_^3−^), chelation, and other dedicated technologies depending on the needs, which are determined, for example, by the physicochemical composition and intended use of the final product [20].

Raw MSWIFA use causes the mixture to swell due to the Al content, which reacts with water at a pH above 9–9.5. The reaction produces volatile hydrogen, which causes the mixture to swell. The swelling of the mixture has a destructive effect on its matrix and lowers its strength. Swelling is seen in the formation of bubbles or swelling across the entire surface of the mix. The MSWIFA pre-treatment allows for the oxidation of hydrogen with Al and Al/Zn [32,33].

The amount of emissions and the carbon footprint depend on many factors, for example, the technology and process of clinker burning or the materials used (e.g., RDF). Another category of environmental impact may be abiotic depletion, calculated in antimony equivalent. For this purpose, you can use tools such as OpenLCA (https://www.openlca.org, accessed on 12 December 2023) or SimaPro software (https://simapro.com, accessed on 12 December 2023) and the Ecoinvent database. However, LCA analysis is not the subject of this article. It is to investigate the impact of MSWIFA as a component of the construction mixture. Global trends indicate the closing of loops, which is consistent with the circular economy, which is the basis for stating that mixtures containing MSWIFA can aspire to be called an eco-product.

## 2. Materials

**Figure 3:**
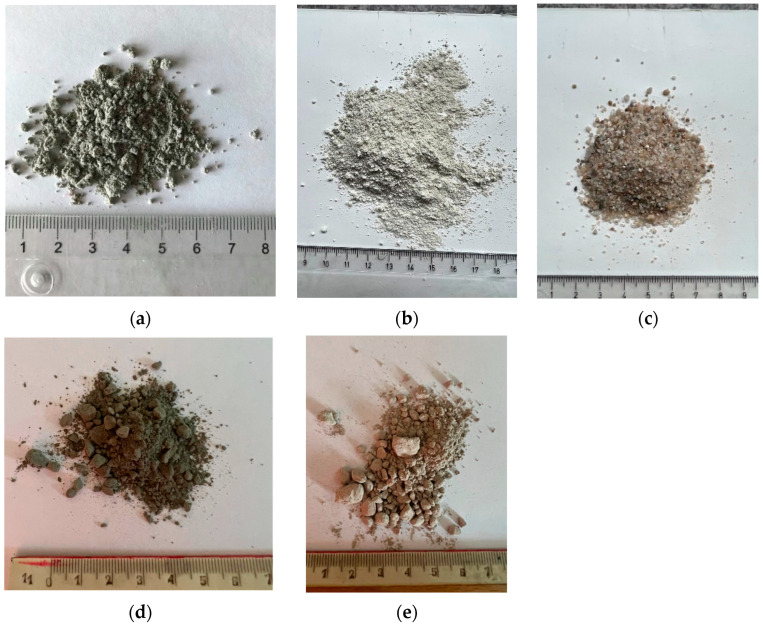
Materials used in the concrete with MSWIFA: (**a**) hazardous fly ash from the municipal solid waste incineration plant (EWC 19 01 07 *); (**b**) zeolite (powder form); (**c**) sand aggregate; (**d**) CEM I 42.5R; (**e**) CSA.

The research material is the by-products of a municipal solid waste incineration plant (European Waste Code: 19 01 07 *). Fly ash is a hazardous, dusty, light gray waste with pozzolanic properties. It is characterized by a high content of heavy metals, chlorine, lime, and sulfur. The leachability of pollutants is exceeded and does not meet the requirements of the Regulation of the Minister of the Environment of 18 July 2014 on the conditions to be met when discharging sewage into water or soil and on substances particularly harmful to the aquatic environment (Journal of Laws, item 1800) and the Regulation of the Minister of Economy of 16 July 2015 on the admission of waste to landfills (Journal of Laws, item 1277). Fly ash is shown in Figure 3a.

Zeolite is a construction additive with immobilizing properties for heavy metals. Its spatial structure makes it possible to keep hazardous waste (and its pollution) inside the concrete structure. Zeolite has pozzolanic properties and increases the viscosity of the mixture. The powder form of clinoptilolite zeolite is used in the mixture, with a density of 2.27 g/cm^3^ and a water requirement of 122% mass (ASTRA Z-50). The zeolite is shown in Figure 3b. Sand is a fine-grained aggregate, and it is shown in Figure 3c.

Figure 3d shows CEM I 42.5 R, which is a highly used material. CEM I 42.5 R has properties such as very low alkali content, high resistance to alkaline corrosion, quick strength gain, high early strength (after 2 days), high strength in the normal period (after 28 days), and stable quality parameters.

Figure 3e shows calcium sulphoaluminate cement (CSA). CSA has properties such as a very short setting time, a very dynamic increase in strength in early adolescence, and less contraction.

The mortars that were the subject of research were composed of binder in the form of ordinary Portland cement (OPC) blended with CSA, CEN standard sand, and tap water. The water/binder ratio was 0.42 to enhance the mechanical and durability properties of the resulting composites. CSA was used to decrease the free CaO content in the cement blend. It was necessary because of the extensive swelling of the mortars. The CSA/OPC ratio was reported to be optimal in the range of 70:30–80:20 [34]. In this research, the CSA/OPC ratio of 63:37 was previously examined and proven to cause the lowest volume changes in cement. The current examinations also prove this. The phase compositions of OPC and CSA cement are given in Table 3. The binder blend was modified with MSWIFA. The research was conducted to establish the possibility of encapsulating it in the cement composites. The MSWIFA was added only in the amount of 5.8% of the binder’s mass, and it is caused by excessive swelling of mortars. Zeolite in powdered form was used to immobilize hazardous compounds from MSWIFA and prevent it from leaking. Several admixtures were used to enhance the properties of the mixture and hardened mortar: an air entraining agent to improve frost resistance, a superplasticizer to compensate for consistency worsened by a low water/binder ratio, and a retarder to override the fast setting of CSA cement and prolong the workability period. The exact compositions of individual mixes are given in Table 4.

## 3. Methods

The roperties of fresh mortars were tested according to EN standards. A list of standards is given in Table 5.

## 4. Results

### 4.1. MSWIFA Heavy Metal Content Determination

The study of heavy metals (Table 6) consisted of mineralizing the MSWIFA sample and then preparing 100 mL of an aqueous solution. The water extract was examined using a flame spectrometer.

The leaching amounts exceeded the allowable values for Class III groundwater. The greatest amount is Pb (907.83 mg/kg). A similar case has been investigated [42].

### 4.2. MSWIFA Leachability Tests

The leachability of pollutants determines the amount of selected substances that are released into the environment. The leachability test was performed by shaking (24 h at 20 °C) in a dark, closed sample container in the presence of distilled water in a ratio of 1:10. The water extract was tested for the content of selected elements. Table 7 shows the leachability of heavy metals from MSWIFA, and Table 8 shows the leachability of the rest of the selected parameters.

In the research sample, Cu is below the limit of quantification, but this is not a rule. The properties of MSWIFA depend on many factors, i.e., season, installation, place, regulation, social habits, etc. [42].

In tased MSWIFA there is considerable leachability of Na (14,820 mg/dm^3^), K 1129 mg/dm^3^, Ca 10,410 mg/dm^3^ and Ba 596 mg/dm^3^.

### 4.3. Consistency of Mortars and Its Maintenance over Time

**Figure 4:**
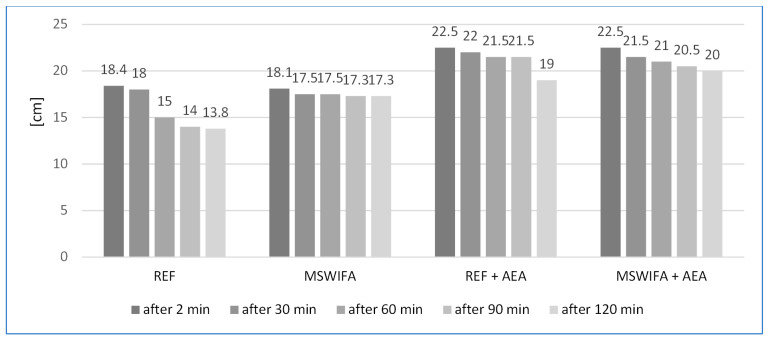
Consistency of mortars immediately after mixing and behavior in time up to 120 min.

The results of consistency measurements for all four types of mortars are presented in Figure 4. The reference one (REF) contained a blend of cementitious materials (Portland and CSA cement), zeolite, water, superplasticizer, and retarding admixture. The flow diameter of this mortar was 18.4 cm immediately after mixing and dropped to 13.8 cm after 2 h. The biggest drop from 18.0 to 15.0 cm was found between 30 and 60 min. The problem of maintaining consistency in time and different temperatures for CSA cements was the subject of some research [43,44]. The second type of mortar also contained Municipal Solid Waste Incineration Fly Ash (MSWIFA) as a partial replacement for cement. The flow diameter of 18.1 cm after mixing was very similar to that of REF mortar. The difference is that it sustained consistency up to 2 h, and the decrease in flow diameter was only 0.8 cm. The reason for this is the regular round shape of ash’s grains, which acts as a bearing mechanism [45,46]. The second reason may be the delaying of the initial setting time, caused by MSWIFA and described below. Two additional mixes (REF + AEA and MSWIFA + AEA) were prepared, both similar to those described above but modified with air entraining agent (AEA). Both exhibited improved consistency in comparison to non-aerated mortars, reaching 22.5 cm of flow diameter immediately after mixing. The effect of AEA on improving consistency is proved by several studies [47,48,49]. In both cases, the decrease in time was visible. The former one, without MSWIFA, showed a drop from 21.5 to 19.0 cm between 90 and 120 min. The latter, containing MSWIFA, decreased consistency more smoothly down to 20.0 cm after 2 h.

All mortars exhibited a long period of workability, enough for seamless application. The factor decreasing the consistency in time is CSA cement, which is characterized by fast setting [50,51,52]. The effect of this rapid behavior was mitigated by both blending it with CEM I and retarding admixture usage.

### 4.4. Air Content and Its Maintenance over Time

**Figure 5:**
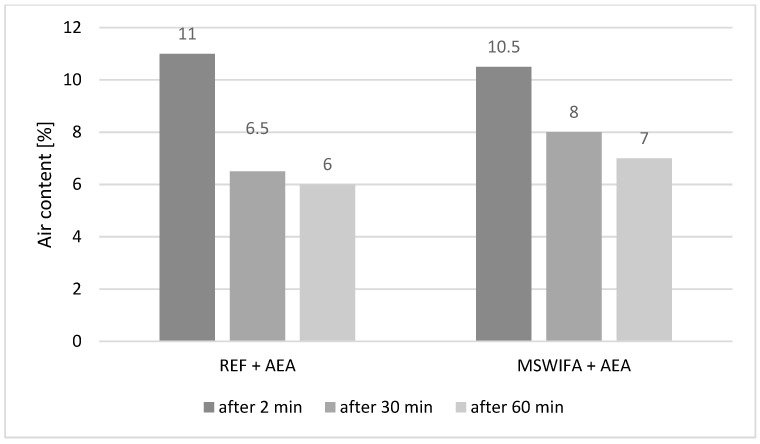
Air content in mortars immediately after mixing and behavior over time up to 60 min.

The air content was tested only for aerated mixtures. The results are given in Figure 5. After mixing both mortars, REF + AEA and MSWIFA + AEA contained similar amounts of air: 11.0 and 10.5%, respectively. This amount corresponds to 5–5.5% of air in concrete [53,54] that contains coarse aggregate and ensures the proper frost resistance of the resulting composite according to EN 206-1 2003 [55]. It is essential to note that reaching this amount of air content required more AEA admixture in the case of mortar containing MSWIFA. This is similar behavior to mortars containing fly ash, which decrease the efficiency of admixtures [56,57,58]. After 30 min, REF + AEA mortar was deaerated down to 6.5%, and the one containing MSWIFA dropped down to 8.0%. This slight difference may be caused by different dosages of AEA to reach the initial air content. After the next 30 min, the decrease in air content was less noticeable, but further delay caused a drop in air content below 6% in mortar, which corresponds to 3% of the air content in concrete mix, which does not ensure frost resistance (the air content required by EN 206-1 is 4%). This issue can be overcome by increasing the dosage of the air-entraining admixture. Such a solution requires further research to verify the effect on mechanical performance.

### 4.5. Initial Setting Time

**Figure 6:**
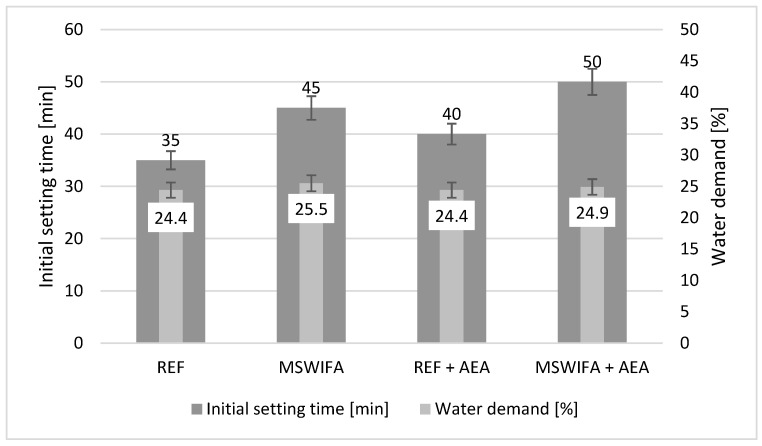
Initial setting time and water demand for cement pastes.

The initial setting time was tested for all mixtures of cement paste. The retarder amount was the same for all mixtures to ensure the accuracy of the results. The results are presented in Figure 6. The initial setting time for REF mortar was 35 min, and the addition of MSWIFA caused its prolongation to 45 min. The most probable cause of such elongation is the zinc content in MSWIFA. Some studies have reported zinc as a retarding and disturbing agent for cement hydration [59,60]. The initial setting time for aerated pastes was slightly longer, but this is a normal effect of aeration [61]. Initial setting time control may be provided by a proper dosage of retarding agent suitable for individual situations and applications.

Disclaimer: The initial setting time and consistency described above (flow table test) sustained over time are tested on different composites (mortar for consistency and cement paste for initial setting time). It is the reason why mortar stays plastic for over 2 h and the initial setting time is lower than 1 h.

### 4.6. Water Demand

Water demand is represented by the percentage of water required for cement paste to reach standard consistency, as specified by EN 196-3. The results are given in Figure 6. The water demand of a blend consisting of Portland cement, CSA cement, and zeolite is 1 percentage point lower than that of this with MSWIFA addition, with the same modification with admixtures. The difference is very low in the case of non-aerated mixtures, and it is in the range of the standard deviation, so it should not be considered meaningful. In the case of aerated pastes, the difference is even smaller. The water demand test corresponds with previous flow table consistency tests for mortars.

### 4.7. Soundness

All mortars were subjected to soundness tests. After testing with Le Chatelier’s apparatus according to the procedure given in the standard EN 196-3:2016, the distance between indicators rose from 10 mm to 14–15 mm, so the difference is 4–5 mm. Since all measured differences are within 10 mm, the binder is suitable for usage in terms of its soundness.

### 4.8. Compressive and Flexural Strength

**Figure 7:**
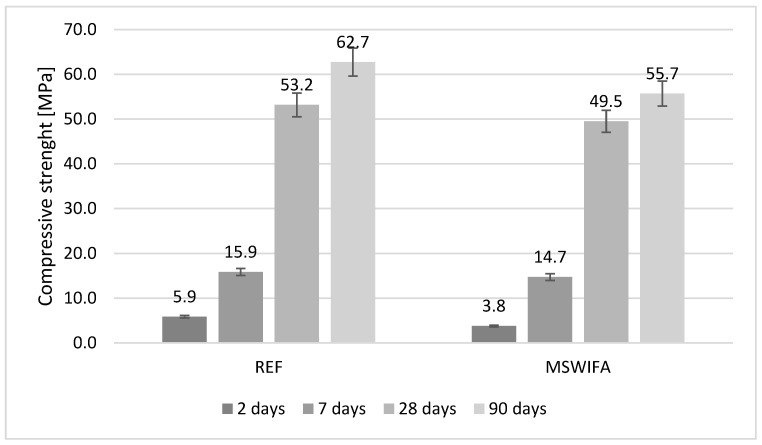
Compressive strength of non-aerated mortars.

**Figure 8:**
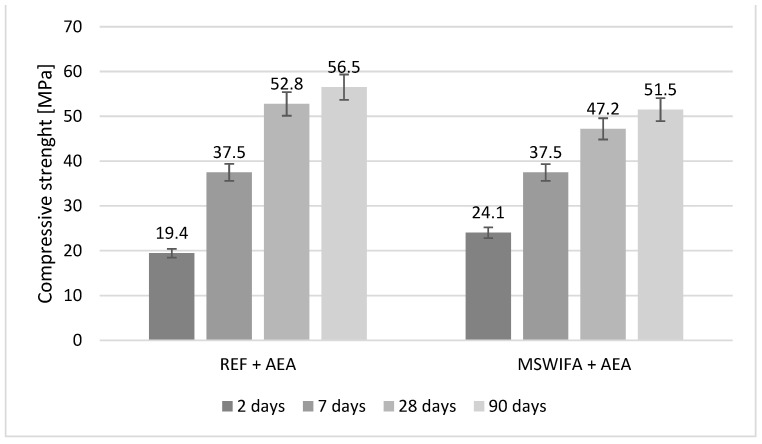
Compressive strength of aerated mortars.

**Figure 9:**
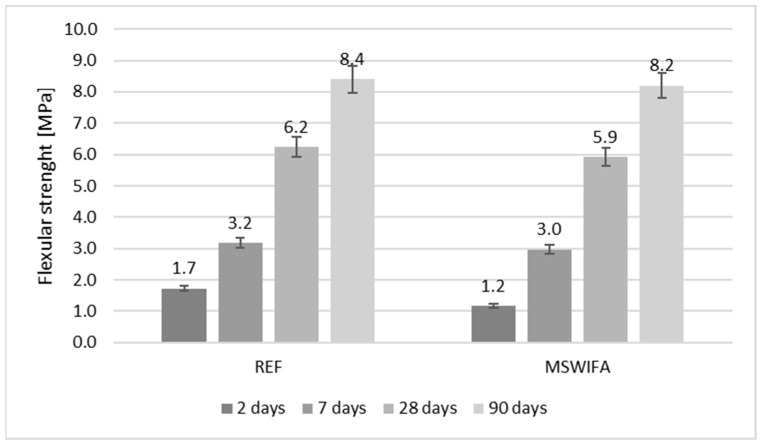
Flexural strength of non-aerated mortars.

**Figure 10:**
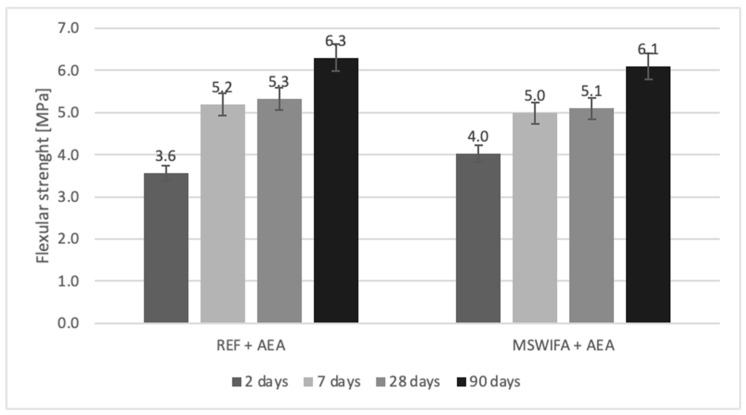
Flexural strength of aerated mortars.

The mechanical parameters were tested and are presented in Figure 7, Figure 8, Figure 9 and Figure 10 for mortars modified with MSWIFA and AEA. The results for aerated and non-aerated mortars should not be compared for early terms (2 and 7 days) because of different retarder dosages. It was necessary to increase the amount of retarder in non-aerated mortars to allow consistency to be maintained over time. After a longer period (28 and 90 days), the effect of retarder is not essential for both compressive and flexural strength; therefore, those values may be compared.

For non-aerated mortars (Figure 7), the compressive strength of the MSWIFA-containing composite is significantly lower after 2 days in comparison to REF mortar. This is the effect of retardation of hydration in the early stage caused by zinc content [59,60]. After 7–90 days, the results are comparable, but still slightly lower for the MSWIFA-containing mortar. It is connected with the presence of MSWIFA, which has no binding properties and acts as a filler material. The behavior of aerated mortars (Figure 8) is similar to visible retardation in the early stages of hardening.

After 28 and 90 days, the decrease in compressive strength is visible for aerated mortars in comparison to non-aerated ones. However, because of the additional air content in such composites, it is worth mentioning this observation [62].

All relationships are similar for flexural strength as well for both non-aerated composites (Figure 9) and aerated ones (Figure 10) in all terms of testing.

### 4.9. Shrinkage

**Figure 11:**
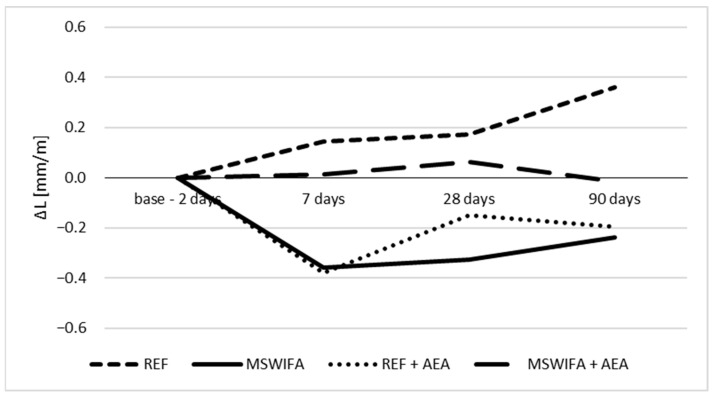
Shrinkage of samples.

Shrinkage examinations were conducted to prove that CSA-CEM I-MSWIFA composites are safe in terms of volume stability. The results presented in Figure 11 differ for different mortars, but all values of shrinkage for every composition of mortar and in every term of testing are below 0.4 mm/m, which is considered in technical specifications as the safe limit (0.52 mm/m, according to ACI 360) to minimize the danger of shrinkage-induced deterioration of concrete. Variations are caused by the inaccuracy of the method and very small relative differences. It is necessary to mention that tests were conducted on mortars, which contain no coarse aggregate, and therefore the shrinkage of concrete containing aggregate and with similar binder compositions will be even lower.

### 4.10. SEM with EDS

CSA cement and its blend with the Portland cement hydration process were subjects of some studies [50,51,52,63]. CSA cement contains ye’elimite and belite as the primary phases available for the hydration process. In the hydration reaction, ye’elimite forms calcium aluminate monosulphate (C_4_ASH_12_) and aluminum hydroxide (AH_3_). In the presence of gypsum, which is a source of calcium sulphate, the product of the hydration reaction is ettringite—calcium aluminate trisulphate (C_6_AŜ_3_H_32_) and aluminum hydroxide (AH_3_). When more calcium hydroxide is available for reaction, only ettringite is formed. Ettringite is responsible for the early strength and reduction of shrinkage (or even expansion) of CSA cement composites. Belite reacts slower, and in the presence of AH_3_, strätlingite (C_2_AŜH_8_) is formed. If AH_3_ is not available, the CSH phase and calcium hydroxide (CH) may be produced as well. CH accelerates the reaction of ye’elimite with water. Belite is responsible for the long-term strength of CSA cements. Portland cement hydration products are mainly CSH phase, calcium hydroxide (CH), and also ettringite in the early stages of hydration.

SEM and EDS analyses were performed for REF and MSWIFA samples. Samples were cured for 28 days in water at 20 °C, then dried and broken. Samples were treated with a thin layer of gold for better transmittance. Peaks of gold on the EDS spectrum are present but removed from the atomic composition tables.

**Figure 12:**
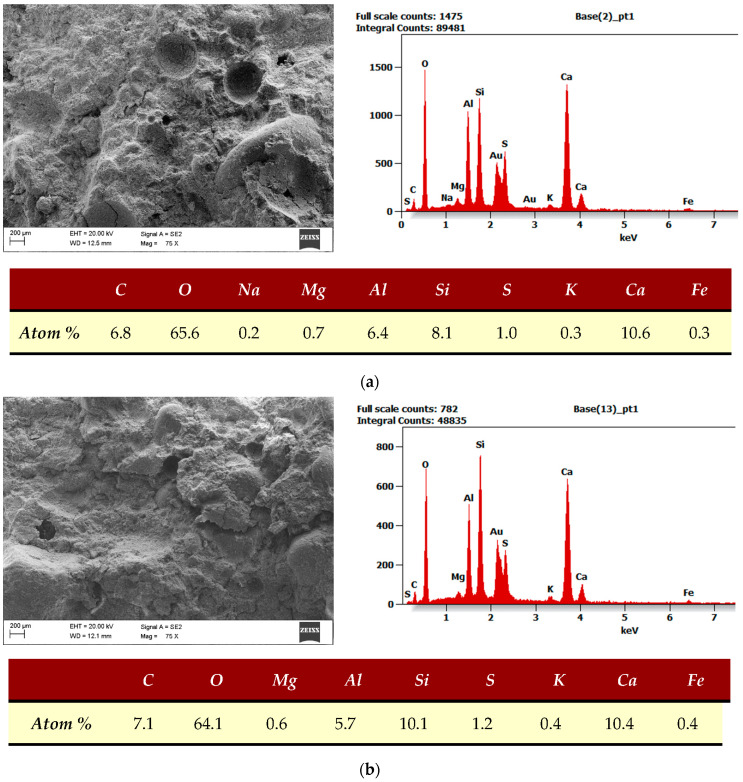
General SEM images and EDS analysis. EDS covers the whole visible area. (**a**) REF sample. (**b**) MSWIFA sample.

Figure 12a is a general view SEM image and EDS analysis of the whole area visible on the SEM image for the sample without MSWIFA. Figure 12b shows the same for the MSWIFA-modified sample. Because of the small amount of MSWIFA, the EDS analysis gave similar results for both samples.

**Figure 13:**
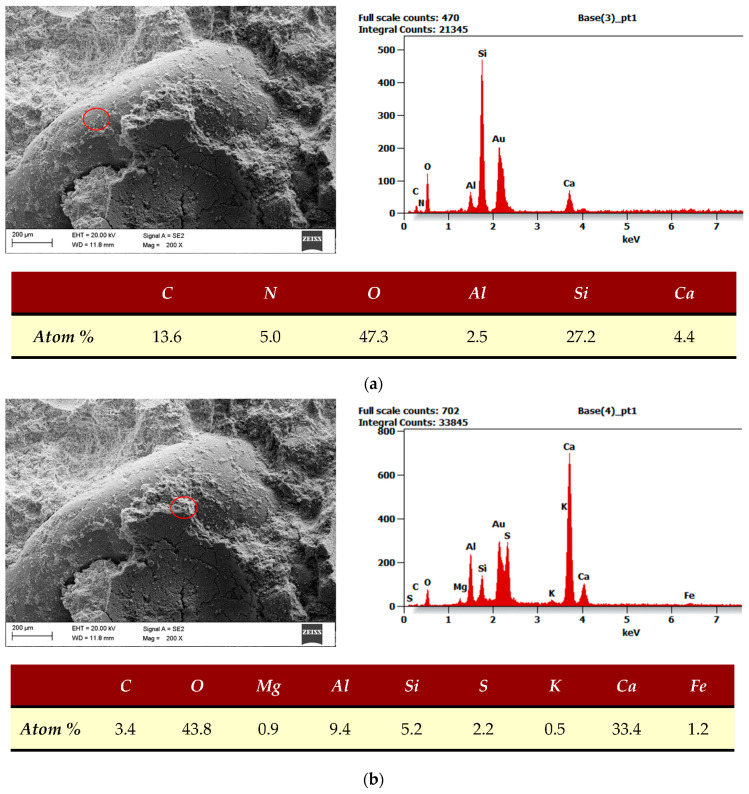

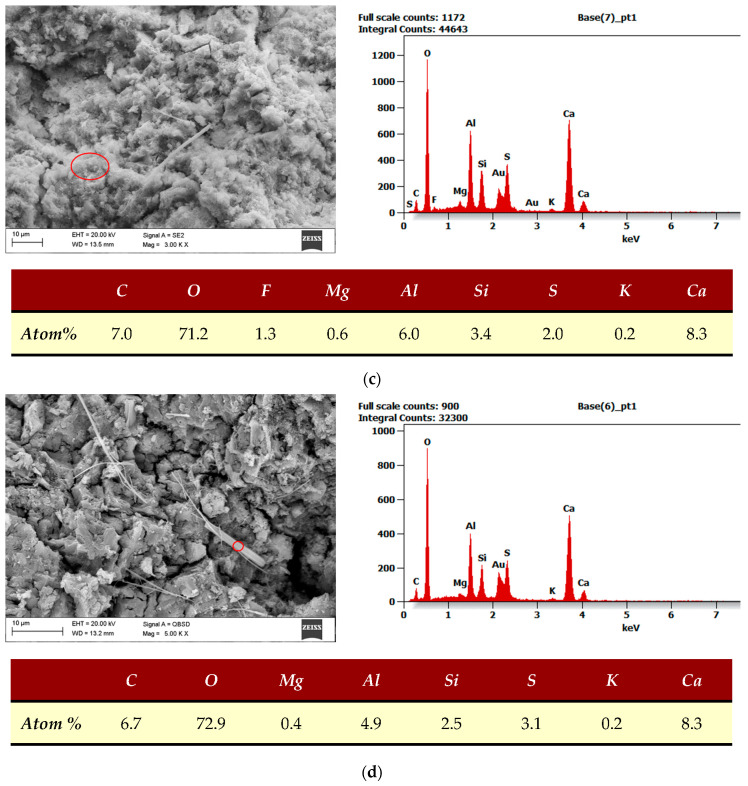
SEM with EDS of reference mortars. (**a**) Sand particles covered with CSH and CASH phases in the REF sample. (**b**) CSH and CASH phases covering sand particles in the REF sample. (**c**) CSH and CASH phases in the REF sample. (**d**) Ettringite in the REF sample.

Because a blend of CEM I and CSA cements was used for the preparation of composites, separate hydration products are hard to distinguish, but an attempt was made. In the reference sample, sand particles coated with CSH and CASH phases are found. Examples are presented in Figure 13a,b. CSH and CASH phases fill most of the sample area (Figure 13c). Some secondarily formed ettringite is also present in the form of thin needles (Figure 13d).

**Figure 14:**
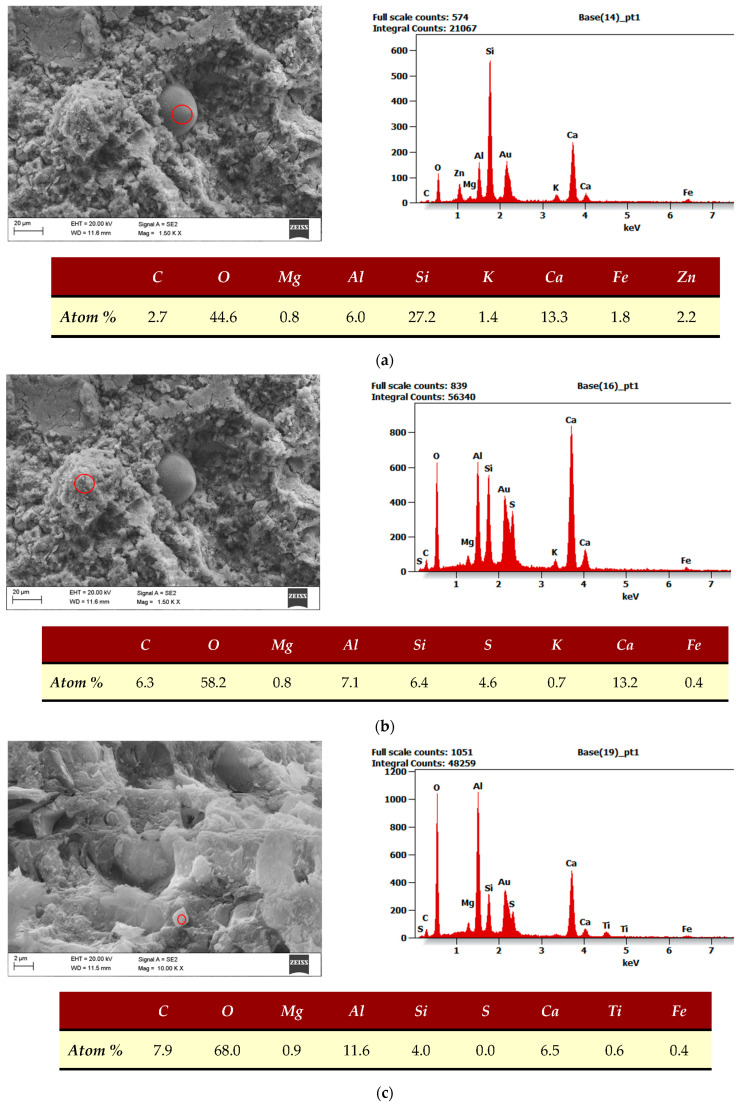

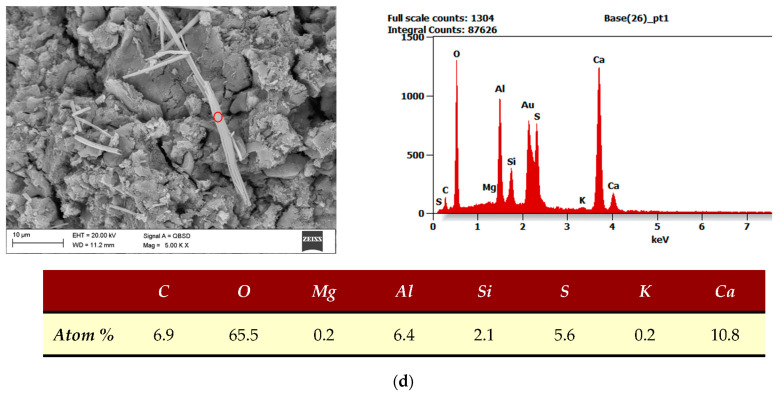
SEM with EDS of mortars with MSWIFA. (**a**) Sand particle and zinc presence in the MSWIFA sample. (**b**) CSH and CASH phases in the MSWIFA sample. (**c**) Titanium traces in the MSWIFA sample. (**d**) Ettringite in the MSWIFA sample.

The sample containing MSWIFA looks similar to the REF sample. It is caused by a small amount of waste, and it is desirable because it proves that the morphology of the cement matrix is not altered by MSWIFA addition. The sand particles and CSH and CASH phases occupy most of the observed area (Figure 14a,b). Some zinc and titanium originated from MSWIFA were found in the sample (Figure 14c,d).

## 5. Conclusions

### 5.1. Market and Environmental Conclusions

Increasingly stringent restrictions and regulations introduced by the member states of the United Nations and the European Union, as well as shrinking natural resources, force the market to look for alternative production solutions in many industries. Growing recycling targets influence the search for innovative ways of waste management. The construction industry is at the forefront of global greenhouse gas emitters, but it can also be the last element in the circular economy. Searching for substitutes for natural raw materials in waste management and replacing them with building materials reduces the amount of waste in landfills and does not extract natural resources to produce building materials from them. Thanks to the closure of the cycle, the life cycle of materials is extended, which is environmentally and economically beneficial.

In the construction industry, the most critical point as an emitter of carbon dioxide is the production of cement. Finding new solutions to replace the binder is economically beneficial due to the reduction of production costs related to the fees for the sale of CO_2_ emission rights. Therefore, a substitute for building material, which is a concrete with MSWIFA blend, on the one hand, reduces environmental costs, including reduction of CO_2_ emissions by giving up cement, and on the other hand, a place to be developed (currently stored in old mines) by MSWIFA.

### 5.2. Summary of the Construction Findings of This Study

The consistency of mortars containing MSWIFA and those without it is similar immediately after mixing. Mortars containing MSWIFA exhibit a slower decrease in consistency over time. All mortars exhibit workability long enough for seamless application.

Air entraining admixture efficiency is lowered by MSWIFA content in composite, but the air content is more stable in time for this mortar in comparison to the reference one.

MSWIFA, because of its zinc content, slightly increases the initial setting time. The same reason is for decreased compressive and flexural strength in the early stages of hardening.

MSWIFA does not influence the water demand and volume stability of mortars. The latter is proved both by a soundness test for cement and a shrinkage test for mortars.

## Figures and Tables

**Table 1:** Chemical compositions of MSWIFA from different sources, %.

Parameter	CaO	SiO_2_	MgO	Al_2_O_3_	FeO_3_	P_2_O_5_	Na_2_O	K_2_O	Cl	Ref.
MSWIFA 19 01 07 *	16.4	27.2	2.5	11.7	1.8	0.3	5.9	5.8	7.2	[23]
	47.4	2.2	1.6	1.0	0.8	0.7	4.9	6.7	25.0	[24]
	45.4	13.6	3.2	0.9	3.8	1.7	4.2	3.9	9.7	[25]
	46.8	8.8	1.6	2.6	0.9	1.2	4.4	4.9	N.P.	[26]
	46.3	8.4	2.7	1.8	1.3	0.7	6.2	5.2	N.P.	[27]

N.P.—not reported.

**Table 2:** Heavy metal content of MSWIFA, mg/kg.

Symbol	Zn	Ba	Cu	Pb	Mn	Ni	Cr	Ref.
Parameter	Zinc	Bar	Copper	Lead	Manganese	Nickel	Chrome	
MSWIFA 19 01 07 *	17,000	140	840	3000	1100	220	490	[28]
	37,384	N.P.	3081	1356	N.P.	1584	566	[29]
	3692	N.P.	2817	826	N.P.	78	1369	[30]
	N.P.	123	405	908	787	46	80	o.r. **

N.P.—not reported. ** own research. Results are posted in the Introduction for comparative purposes. The description of the study is discussed in the Section 4.

**Table 3:** Phase composition of Portland clinker (OPC) and calcium sulphoaluminate (CSA) clinker.

Clinker	Composition [%]								
	C_3_S (Alite)	C_2_S (Belite)	C_3_A (Celite)	C_4_AF (Brown Millerite)	C_4_A_3_Ŝ (Ye’Elimite)	CŜ (Anhydrite)	3C_2_S·3CŜ·CaF_2_ (Fluorellastidite)	MgO (Periclase)	C_3_MS_2_ (Merwinite)
CSA	-	10.4	-	1.2	64.9	2.6	9.4	4.9	0.8
OPC	69	9.6	9.4	9	-	-	-	0.15	-

C—CaO; S—SiO_2_; A—Al_2_O_3_; F—FeO; Ŝ—SO_3_; M—MgO.

**Table 4:** Composition of mortars [g].

	Reference Sample	Municipal Solid Waste Incineration Plant Fly Ash Sample	Reference Sample + Air-Entraining Admixture	Municipal Solid Waste Incineration Plant Fly Ash Sample + Air-Entraining Admixture
CEM I 42.5 R	108	100	108	100
CSA	284	270.5	284	270.5
Zeolite	60	60	60	60
MSWIFA	-	21.5	-	21.5
Sand *	1350	1350	1350	1350
Water	190	190	190	190
SPF	13.5	13.5	13.5	13.5
Retarder	13.56 (10.30) **	13.56 (10.30) **	10.3	10.3
air-entraining admixture (AEA)	-	-	0.04	0.1

* for initial setting time, soundness, and water demand, cement paste was used without the sand; ** (10.30) was used for the initial setting time test only for comparison with aerated composites.

**Table 5:** Examined properties and research methods.

Property	Standard/Method
Water extract	Water extract was prepared according to EN 12457-2:2006 [35]. Sample was shaken in water for 24 h in a 1:10 proportion (sample/water).
Heavy metal content	Heavy metal content was determined according to EN 16174:2012 [36] and EN ISO 11885:2009 [37].
Consistency	EN 1015-3:2000/A2:2007 [38] Methods of testing mortar for masonry—Part 3: determination of the consistency of fresh mortar (by flow table).
	The consistency was tested just after mixing and after 30, 60, 90, and 120 min. Before each test, the mortar was mixed for 15 s in the mixer.
Air content	EN 1015-7:1998 [39] Methods of testing mortar for masonry—Part 7: Determination of air content of fresh mortar.
	The air content was tested just after mixing and the after 30 and 60 min
Water demand	EN 196-3:2018 [40] Methods of testing cement—Part 3: Determination of setting times and soundness.
Initial setting time	
Soundness	
Flexural and compressive strength	EN 196-1:2018 [41] Methods of testing cement—Part 1: Determination of strength.
	The samples in the form of prisms measuring 160 × 40 × 40 mm were molded and kept in a humidity of 90% and a temperature of 20 ± 1 °C for 24 h. Then, samples were demolded and placed in the water at 20 ± 1 °C. Both flexural and compressive strengths were tested after 2, 7, 28, and 90 days.
Shrinkage	The samples in the form of prisms measuring 160 × 40 × 40 mm, equipped with metal tips on both ends, were molded and kept in a relative humidity of 90% and a temperature of 20 ± 1 °C for 24 h. Then samples were demolded and put in the climatic chamber at 20 ± 1 °C and 60% relative humidity. The elongation/shortening of the samples was tested. The first measurement was conducted after 48 h, and it was used as a base for further measurements after 7, 28, and 90 days.
SEM, EDS	The examination was performed using a scanning microscope (Supra35 Zeiss with EDS detectors and WDS–EDAX from EDAX Company, Mahwah, NJ, USA). The measurement was based on the measurement of the characteristic X-ray energy spectrum. The research was done in The Silesian University of Technology in Gliwice.

**Table 6:** Heavy metal content of MSWIFA.

Symbol	Ba	Cu	Pb	Mn	Ni	Cr	Cd	Co
Parameter	Barium	Copper	Lead	Manganese	Nickel	Chromium	Cadmium	Cobalt
Unit	mg/kg							
Results	123.10	404.82	907.83	787.10	46.47	80.13	98.81	10.04

**Table 7:** Heavy metal leachability.

Material	Cadmium	Chrome	Copper	Iron	Manganese	Nickel	Lead	Zinc
Symbol	Cd	Cr	Cu	Fe	Mn	Ni	Pb	Zn
Unit	µg/mL	µg/mL	µg/mL	µg/mL	µg/mL	µg/mL	µg/mL	µg/mL
MSWIFA 19 01 07 *	0.02189	0.77382	BLQ	BLQ	BLQ	0.18530	1.42056	45.8850

Below the limit of quantification.

**Table 8:** Fly ash leachability of selected parameters.

Element	Symbol	Unit	MSWIFA 19 01 07 *
Sodium	Na	mg/dm^3^	14,820.0
Potassium	K	mg/dm^3^	1129.0
Lithium	Li	mg/dm^3^	10.0
Calcium	Ca	mg/dm^3^	10,410.0
Barium	Ba	mg/dm^3^	596.0

## Data Availability

Data are contained within the article.
